# Implications of shared predation for space use in two sympatric leporids

**DOI:** 10.1002/ece3.4980

**Published:** 2019-02-20

**Authors:** Martijn J. A. Weterings, Sophie P. Ewert, Jeffrey N. Peereboom, Henry J. Kuipers, Dries P. J. Kuijper, Herbert H. T. Prins, Patrick A. Jansen, Frank van Langevelde, Sipke E. van Wieren

**Affiliations:** ^1^ Resource Ecology Group Wageningen University Wageningen The Netherlands; ^2^ Department of Animal Management, Wildlife Management Van Hall Larenstein University of Applied Sciences Leeuwarden The Netherlands; ^3^ Mammal Research Institute Polish Academy of Sciences Białowieża Poland; ^4^ Smithsonian Tropical Research Institute Balboa Panamá; ^5^ School of Life Sciences University of KwaZulu‐Natal Durban South Africa

**Keywords:** alternative prey, habitat characteristics, habitat riskiness, residence time, space race, vegetation structure

## Abstract

Spatial variation in habitat riskiness has a major influence on the predator–prey space race. However, the outcome of this race can be modulated if prey shares enemies with fellow prey (i.e., another prey species). Sharing of natural enemies may result in apparent competition, and its implications for prey space use remain poorly studied. Our objective was to test how prey species spend time among habitats that differ in riskiness, and how shared predation modulates the space use by prey species. We studied a one‐predator, two‐prey system in a coastal dune landscape in the Netherlands with the European hare (*Lepus europaeus*) and European rabbit (*Oryctolagus cuniculus*) as sympatric prey species and red fox (*Vulpes vulpes*) as their main predator. The fine‐scale space use by each species was quantified using camera traps. We quantified residence time as an index of space use. Hares and rabbits spent time differently among habitats that differ in riskiness. Space use by predators and habitat riskiness affected space use by hares more strongly than space use by rabbits. Residence time of hare was shorter in habitats in which the predator was efficient in searching or capturing prey species. However, hares spent more time in edge habitat when foxes were present, even though foxes are considered ambush predators. Shared predation affected the predator–prey space race for hares positively, and more strongly than the predator–prey space race for rabbits, which were not affected. Shared predation reversed the predator–prey space race between foxes and hares, whereas shared predation possibly also released a negative association and promoted a positive association between our two sympatric prey species. Habitat riskiness, species presence, and prey species’ escape mode and foraging mode (i.e., central‐place vs. noncentral‐place forager) affected the prey space race under shared predation.

## 1 INTRODUCTION

**Figure 1:**
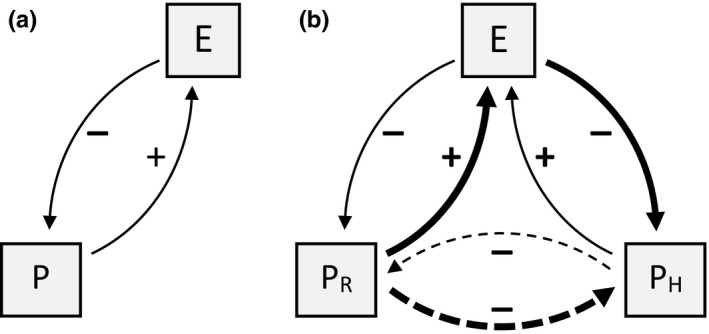
Conceptual representation of the relationship between space use by prey and that of their predators: (a) single prey predator system with prey (P) and predator (E), (b) predator two‐prey system with asymmetrical apparent competition between two prey species (P_R_ = rabbit; P^H^ = hare) that share a common predator (i.e., red fox) (adjusted from Chaneton & Bonsall, 2000). Solid lines are direct effects; dashed lines are indirect effects. Arrows point toward dependent entity. Negative magnitudes indicate spatial avoidance by prey; positive magnitudes indicate spatial aggregation by predator. Arrow widths indicate the relative strength of the effects

In the behavioral response race between predators and their prey (Sih, 2005), predators select locations and times in response to the use of space by prey (Laundré, 2010), resulting in “risky places” and “risky times” (Creel, Winnie, Christianson, & Liley, 2008, i.e., landscape of fear, Laundré, Hernández, & Altendorf, 2001), whereas prey in turn often select locations and times to avoid these risky places and times (Lima & Dill, 1990) (Figure 1a). The predator–prey space race, based on game theory, is affected by fear imposed by the habitat or the “inherent riskiness of the habitat” (sensu Hugie & Dill, 1994), which is determined by the habitat characteristics that affect the probabilities of attack and escape (Bednekoff & Lima, 1998).

The habitat characteristics that determine the probability of an attack (i.e., pre‐encounter risk) are not necessarily the same habitat characteristics that determine the probability of escape from a predator (i.e., postencounter risk) (Gorini et al., 2012). For example, open habitats with low vegetation density, little cover, or short vegetation provide high visibility for the prey and facilitate movement or escape (Gorini et al., 2012; Lima, 1992), however, they have low possibilities for hiding and concealment. In contrast, more closed habitats have low visibility for the prey and hinder movement or escape, however, they have more possibilities for hiding and concealment (Wirsing, Cameron, & Heithaus, 2010). As a result, spatial variation in habitat riskiness has a major influence on the relation between the use of space by prey and predators (Chesson, 2000); however, its outcome can be modulated if prey shares enemies with fellow prey (i.e., another prey species).

Sharing of natural enemies by different prey species is common in natural communities (Chaneton & Bonsall, 2000). It may result in apparent competition, an indirect effect in which a given prey species experiences more predation risk resulting from the presence of fellow prey (Holt, 1977, 2009). Competition for enemy‐free space causes prey species to avoid risky places and times as a result of the presence of fellow prey, that is, short‐term apparent competition or aggregative response (Holt & Kotler, 1987; Holt & Lawton, 1994). The effect of a shared predator on each prey species, among others, depends on the amount of resource overlap and spatial overlap between prey species, and the riskiness of the habitat (DeCesare, Hebblewhite, Robinson, & Musiani, 2010). Additionally, differences in prey escape strategy (e.g., the use of refuges), predator type, and the density of additional prey and predators that influence the probability of an encounter and/or the subsequent probability of an attack can affect the response of prey to shared predation (Carbone & Gittleman, 2002; Holley, 1993).

However, the implications of shared predation on prey space use remain poorly studied (Chaneton & Bonsall, 2000). In particular, more insight is needed on the fine‐scale space use by prey and predator species as a result of the interaction between habitat riskiness and the presence or absence of shared predation (see e.g., Camacho, Sáez‐Gómez, Potti, & Fedriani, 2017). Spatial variation in habitat riskiness, for example, can prevent depensatory predation and extinction of prey (Sinclair et al., 1998). However, the effect of spatial variation in habitat riskiness on the relation between the use of space by prey that share a predator is not well understood (DeCesare et al., 2010; Oliver, Luque‐Larena, & Lambin, 2009; Wirsing et al., 2010). Therefore, our objective was to test how prey species spend time among habitats that differ in riskiness, and how shared predation modulates the space use by prey species.

## 2 STUDY SYSTEM

We used a one‐predator, two‐prey system composed of the European hare (*Lepus europaeus*, Pallas, 1778) and European rabbit (*Oryctolagus cuniculus*, Linnaeus, 1758) as sympatric prey species and the red fox (*Vulpes vulpes*, Linnaeus, 1758) as their main predator. We used European hare as the focal species and rabbit as the fellow prey species and vice versa to investigate prey space use. Rabbits are social central‐place herbivores and prefer edge habitat near grasslands (Bakker, Reiffers, Olff, & Gleichman, 2005; Barnes & Tapper, 1986), such as coastal dune habitat. The hare is a solitary noncentral‐place herbivore that is common in open grassland areas (Barnes & Tapper, 1986), such as agricultural (Smith, Jennings, & Harris, 2005), and coastal habitat (Kuijper & Bakker, 2008). Although there is considerable spatial overlap in habitat use between rabbits and hares (Flux, 2008), their habitat‐specific escape modes differ markedly. Rabbits use their burrows to escape predation risk. According to Bakker et al. (2005), rabbit space use is not affected by habitat riskiness or predation risk. In contrast, the effect of hare predation has been suggested to depend on the available vegetation structure, cover, and openness of the landscape (Focardi & Rizzotto, 1999; Smith et al., 2005). Hares are known to use tall vegetation as cover or resting places (Neumann, Schai‐Braun, Weber, & Amrhein, 2011). Additionally, hare space use is sometimes positively or negatively related to edge habitats (Bresinski, 1983; Caravaggi, Montgomery, & Reid, 2015), presumably depending on the associated riskiness of the habitat. Hares stand up to predators and can make use of crypsis and flight (Focardi & Rizzotto, 1999). Foxes can substantially impact hare and rabbit populations (Banks, 2000; Schmidt, Asferg, & Forchhammer, 2004); however, foxes often prefer rabbits over other species (Díaz‐Ruiz et al., 2011; Norbury, 2001; Smith & Quin, 1996). Red fox is known to make use of linear landscape features (Frey & Conover, 2006), and select for ecotones and habitats with protective cover (Kiener & Zaitsev, 2010) to ambush prey (Holley, 1993); however, foxes can also make use of open areas. We hypothesize that the habitat riskiness perceived by prey species is low in habitats in which predators are inefficient in searching or capturing prey species, such as structurally complex habitats (Hugie & Dill, 1994). Therefore, we expect prey species to perceive low risk in habitats with protective cover (e.g., areas with tall shrubs and half‐open vegetation structures). Besides, we expect prey species to perceive low risk in nonedge habitats, because foxes are efficient in capturing (i.e., ambush) mobile prey using cover that can be associated with edges (Holley, 1993). Even though there is a lack of knowledge on species edge responses, edge habitats affect perceived predation risk by prey species differently than nonedge habitats, because many terrestrial predators probably hunt more effectively along habitat edges, increasing predation (Lesmeister, Nielsen, Schauber, & Hellgren, 2015; Lidicker, 1999; Tscharntke et al., 2012).

The high‐risk open vegetation structures provide quality foraging ground for hares and rabbits (Kuijper & Bakker, 2008). Hares and rabbits have a considerable resource overlap (Kuijper, Wieren, & Bakker, 2004), and Homolka (1987) considered them as competitors when sympatric. Rabbits maintain high‐quality patches with short vegetation (Bakker et al., 2005). Intense grazing by rabbits can change the plant species composition, vegetation height, and perceived predation risk, thereby affecting the interaction between hares and rabbits (Bakker, Olff, & Gleichman, 2007; Shipley, 2007). For example, a change in the plant species composition could lead to an increase or decrease in preferred food plants available for hares (Kuijper & Bakker, 2008; Whinam, Fitzgerald, Visoiu, & Copson, 2014). Moreover, the interspecific interaction between hares and rabbits may also be affected by differences in body size, feeding style, digestive system, and morphology (Bell, 1971; Prins & Olff, 1998). Hares are twice as large as rabbits and have a relatively larger bite size. In comparison to rabbits, hares forage less efficiently on short vegetation and require taller vegetation to obtain their absolute daily energy requirements (Shipley, 2007). Habitat modification by rabbits could reduce hare foraging efficiency, leading to exploitative competition. The smaller rabbits may thus have a competitive advantage over hares in terms of resource exploitation (Persson, 1985), because they are central‐place foragers that are more ecologically specialized (Flux, 2008; Shipley, 2007).

Because of the stronger competitive ability of the rabbit and the ability of the rabbit to use its burrow as a predator‐free space, we hypothesize that the effect of shared predation on the space use by the two prey species is asymmetric in favor of the rabbit (i.e., apparent competition). Such asymmetric indirect effects are often observed in one‐predator, two‐prey systems when prey species differ in prey characteristics (Chaneton & Bonsall, 2000). Therefore, we expect that the indirect effects of rabbits on hares are stronger than the indirect effects of hares on rabbits (Figure 1b). Hence, we investigated the following hypothesis: space use by hares is more strongly negatively affected by shared predation than space use by rabbits.

## 3 METHODS

**Figure 2:**
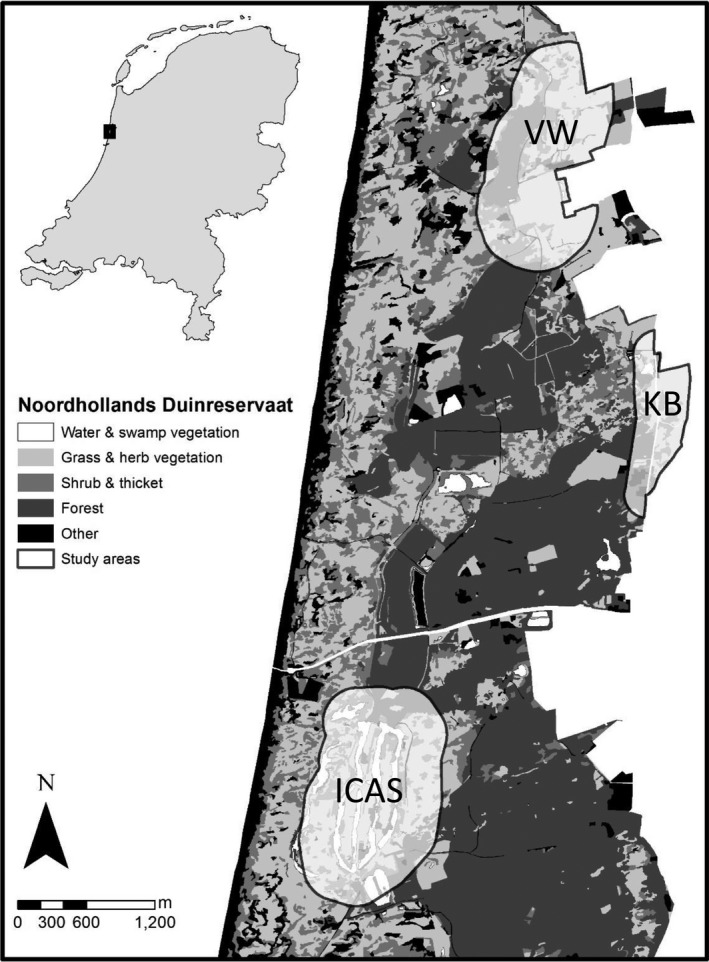
Location of the three study areas with fox, hare, and rabbit populations: Vennewater (VW), Koningsbos (KB), and Infiltration area Castricum (ICAS)

We conducted field work in the coastal dune landscape “Noordhollands Duinreservaat” near Castricum (52°33′N, 4°38′E) in the Netherlands. There were three study areas with small populations of hares and rabbits (Vennewater (VW), Koningsbos (KB), and Infiltration area Castricum (ICAS), Figure 2). We selected the study sites based on previous sightings of hares and rabbits. The study area contained 13 different vegetation types (Supporting Information Table S1) and a mosaic of vegetation, including patches of dune grass, thicket, brushwood, and forest. This late succession vegetation situated on fertile soils, enriched by atmospheric nitrogen deposition, has a high biomass productivity, but parts remain open dune grassland vegetation because of grazing by rabbits and cattle (Kooijman, Dopheide, Sevink, Takken, & Verstraten, 1998). Compared to other areas (Trewhella, Harris, & McAllister, 1988), the fox density in this coastal dune landscape was estimated to be very high, between five and eight individuals per square kilometer (Mulder, 2005).

### 3.1 Field measurements

#### 3.1.1 Space use and habitat riskiness

To study space use, we distinguished four vegetation strata that were related to habitat riskiness. First, we made a distinction between homogeneous patches of open (>90% cover of vegetation with an average herb height <30 cm) and half‐open (≥10% cover of vegetation with an average herb height ≥30 cm) vegetation structures. Open vegetation structures with low vegetation height provide quality foraging ground for hares and rabbits (Kuijper & Bakker, 2008), but little cover, whereas half‐open vegetation structures provide visual cover from predators. Cover is provided when the vegetation is higher than the approximate height of the prey species (i.e., >30 cm for hares) (Neumann et al., 2011). We choose to use a threshold of 10% cover to separate open areas from half‐open area, as especially habitats that have a less dense cover (i.e., <10%, such as pasture) provide little visual cover for predators during the day (Neumann et al., 2011). We considered prey species to perceive low risk in half‐open vegetation structures. Second, we categorized each patch as an edge or nonedge location. We considered prey species to perceive low risk in nonedge locations. Edge habitat was defined as a 12.5‐m strip parallel to a boundary between adjacent (contiguous) vegetation communities that are used differently by prey species, that is, open, half‐open, shrub, and forest (Lidicker, 1999). We chose these dimensions, because (a) obligate rabbit burrowers experience a change in risk over distances less than 10 m (Crowell et al., 2016), (b) hare flight distance is on average less than 12 m (Neumann et al., 2011; Rizzotto & Focardi, 1997), and (c) camera traps are able to detect space use by our species at a maximum distance of 12.5 m.

During five sessions of approximately 15 days each between 16 October 2014 and 8 January 2015, we randomly placed forty‐two cameras (Reconyx Hyperfire: HC500 & HC600, infrared trigger) within the four strata (210 random points, 3,038 successful trap days). The research period was chosen to fall outside of the reproduction period for both prey species to eliminate factors that could cofound space use. Before placement, we took a random sample of possible locations of camera traps from a high‐resolution (1:5,000) GIS map from Everts, Pranger, Tolman, and Vries (2008), Everts, Pranger, Tolman, and Vries (2009). The final locations of the camera traps were interspaced on average by 689 m (*SD* ± 1,189, *n* = 135, range: 4–5,580 m) (Supporting Information Table S3), including >25 m from waterbodies and >16 m from recreational paths. We subdivided each patch near an edge location into three different types depending on the surrounding vegetation (forest, thicket, half‐open, or open) reflecting differences in movement speed. During placement, we positioned cameras at edge locations on the edge itself, facing perpendicularly away from the edge. We directed cameras at nonedge locations north to avoid overexposure by sunlight during the day. Cameras were mounted on a wooden pole, and the line of sight measured from the camera‐lens was calibrated to run parallel to the soil surface at a height of 30 cm up to at least 5 m (*cf* Jansen, Forrester, & McShea, 2014), without clearing any vegetation. In front of each camera, we measured the shrub height in five locations in a 12.5 × 12.5 m orthogonal layout, that is, related to the maximum detection distance. We considered prey species to perceive low risk in patches with tall shrubs. Cameras were set to record a burst of 10 photos (1 s^−1^) when triggered, without any time lapse between bursts.

We quantified residence time by hares, rabbits, and foxes as an index of fine‐scale space use (*T*). We visually assessed residence time of a visit from sequences of camera trap photos. Sequences of trap photos without a quiet period longer than 120 s were defined as visits. Because the average visit of hares, rabbits, and foxes was much shorter than 120 s (Supporting Information Table S3), this seemed justified. We calculated average residence time per visit as a prey response to predation risk (e.g., Fortin et al., 2009; Visscher, Merrill, & Martin, 2017), because in contrast to the total residence time, the average residence time is independent of the frequency a species visits a camera location. The average prey residence time thus differentiates between many quick visits (i.e., high risk) and several longer visits (i.e., low risk) that could add up to the same total residence time. In a similar fashion, the average predator residence time differentiates between many quick visits (i.e., low risk) and several longer visits (i.e., high risk). We corrected residence time for the effective detection area, total deployment time of each camera and speed (Equation ((1))).

Average residence time (s hr^−1^ m^−2^):

(1) $$T_{\text{a}}=\frac{t_{\text{avg}}}{{\left(r_{\text{eff}}\right)}^{2}\left(\frac{\theta_{\text{eff}}}{2}\right)t_{\text{dep}}v_{\text{s}}}$$

*t*_avg_ = average time spent active in front of a camera per visit (s); *r*_eff_ = effective detection distance in open or half‐open vegetation structures (m), *θ*_eff_ = effective detection angle in open or half‐open vegetation structures (rad), *t*_dep_ = deployment time of the camera (hr), *v*_s_ = average geometric speed of animals in each habitat type, relative to the average geometric speed in open or half‐open vegetation structures.

For each species, we measured the detection distance and detection angle of the first capture just before relocating each camera using a wild game viewer. For each species, we estimated the effective detection distance and angle in open and half‐open vegetation structures using R and functions for fitting standard linear covariate detection models to the position of the first capture by camera traps (Rowcliffe, Carbone, Jansen, Kays, & Kranstauber, 2011; Supporting Information Table S2). The effective detection distance and angle defined the area of the habitat type that was surveyed. We measured the distance covered by individuals by a tape measure in front of the cameras, and this distance was used to calculate the average geometric speed of the species in all habitat types (Rowcliffe et al., 2011). We used the average geometric speed of each species in each habitat type relative to the average geometric speed in open or half‐open vegetation structure to correct for the differences in the effective detection areas.

### 3.2 Data analysis

We investigated prey space use by selecting camera locations that captured the species under investigation on at least one occasion. Furthermore, we selected datasets with camera locations that captured either rabbit or hare only to compare prey space use with and without shared predation. This resulted in six nonoverlapping datasets: hare only (21 cameras, 71 visits), rabbit only (36 cameras, 222 visits), hare and rabbit (8 cameras, 258 visits), hare and fox (18 cameras, 186 visits), rabbit and fox (55 cameras, 995 visits), and hare, rabbit, and fox (20 cameras, 1,065 visits). For each species in each dataset, we calculated the average residence time and per camera. Potential effects of undetected foxes were considered negligible, because camera detection correlates positively with body mass and average residence time in front of a camera (Rowcliffe et al., 2011). Effects of species avoiding camera patches, however closely present to a camera, were also considered negligible, because we investigated fine‐scale effects of predation risk in front of the camera (±10–25 m^2^), whereas rabbits and hares experience a change in risk over distances smaller than 12 m (Crowell et al., 2016; Neumann et al., 2011; Rizzotto & Focardi, 1997). Additionally, we assumed that the residence time was not greatly influenced by individuals visiting multiple camera locations (i.e., spatial autocorrelation), because the average distance between camera traps during sessions was 689 m (*SD* ± 1,189, *n* = 135). An overview of the characteristics of the response and predictor variables used in the datasets can be found in the Supporting Information Table S3. Note that the range of habitat characteristics that determine habitat riskiness varies over the datasets.

To assess the effects of predator space use and habitat riskiness on prey space use, we tested the effects of (a) the average residence time of the predator, (b) habitat riskiness (i.e., open or half‐open vegetation structure, edge or noneedge location, and shrub height), and (c) their interactions on the prey average residence time. We hypothesized that hares and rabbits perceived a high risk when fox average residence time increased (Lima & Dill, 1990) (see overview hypotheses in Table 1). Moreover, we hypothesized that risk associated with fox average residence time depended on the habitat riskiness (i.e., context dependent; Kuijper, Bubnicki, Churski, Mols, & Hooft, 2015).

**Table 1:** Overview of hypotheses tested

No.	Hypothesis	Justification	References
1	Habitat riskiness perceived by prey species is low in habitats in which predators are inefficient in searching or capturing prey species such as structurally complex habitats	Closed habitats have more possibilities for hiding and concealment. We expect prey species to perceive low risk in habitats with protective cover (e.g., areas with tall shrubs and half‐open vegetation structures).	Hugie and Dill (1994) and Wirsing et al. (2010)
		We expect prey species to perceive low risk in nonedge habitats, because foxes are efficient in capturing (i.e., ambush) mobile prey using cover that can be associated with edges. Edge habitats affect perceived predation risk by prey species differently than nonedge habitats, because many terrestrial predators probably hunt more effectively along habitat edges, increasing predation.	Holley (1993), Lidicker (1999), Tscharntke et al. (2012), and Lesmeister et al. (2015)
2	The effect of shared predation on the space use by hare and rabbit is asymmetric in favor of the rabbit, that is, space use by hares is more strongly negatively affected by shared predation than space use by rabbits.	Intense grazing by rabbits can change the plant species composition, vegetation height, and perceived predation risk, thereby affecting the interaction between hares and rabbits	Bakker et al. (2007) and Shipley (2007)
		Hares are twice as large as rabbits and have a relatively larger bite size. In comparison to rabbits, hares forage less efficiently on short vegetation and require taller vegetation to obtain their absolute daily energy requirements	Shipley (2007)
		In contrast to hares, rabbits can use a refuge (i.e., burrow) as a predator‐free space	
		Asymmetric indirect effects are often observed in one‐predator, two‐prey systems when prey species differ in prey characteristics	Chaneton and Bonsall (2000)
3	Hares and rabbits perceived a high risk when fox average residence time increased	Prey perceives more risk when a predator spends on average more time in a patch	Lima and Dill (1990)
4	Risk associated with fox total residence time was depended on the habitat riskiness (i.e., context dependent)	Predation risk and habitat riskiness interact to affect prey response	Kuijper et al. (2015)
5	Hares and rabbits perceived a high risk with an increase in the fellow prey average residence time	Prey perceives more risk when fellow prey that increase predation risk spends on average more time in a patch	Holt (2009)
6	Risk associated with fellow prey average residence time, depended on predator space use and the habitat riskiness (i.e., context dependent)	Predation risk and habitat riskiness interact to affect prey response	Kuijper et al. (2015)

To compare datasets and assess whether and how shared predation affected prey space use, we additionally tested the effects of (a) the average residence time of fellow prey, (b) the interaction between the average residence time of fellow prey and predators, and (c) the interaction between the average residence time of fellow prey and habitat riskiness on the prey average residence time. We hypothesized that hares and rabbits perceived a high risk with an increase in the fellow prey average residence time that can increase predation risk for prey with a shared predator (Holt, 2009). Moreover, we hypothesized that risk associated with fellow prey average residence time depended on predator space use and the habitat riskiness (Kuijper et al., 2015).

We assessed the prey average residence time by running linear mixed models (lmer, R Package lme4 version 1.1‐12). Models were kept simple (a maximum of three parameters, i.e., two main effects and one interaction; 2^number of parameters^ ≤ number of cameras) because of the low number of cameras in each dataset. We assessed multicollinearity of continuous predictor variables by a script from Zuur, Ieno, and Elphick (2010). The variance inflation factor (VIF) of all continuous predictor variables remained below 1.9 in all datasets, except for dataset B (i.e., hare and rabbit), in which the average residence time of rabbits was highly correlated with shrub height. Each model included the session number as a random factor and was weighted according to the square root of the number of photos of the response species taken at a camera location (Lipták, 1958). We could not use IT criteria (i.e., AICc) to compare models in different datasets, because the selected datasets contained different samples, that is, not all cameras where visited by all species studied. We thus assessed the relative strength of the parameters using standardized regression coefficients. The hare, rabbit, and fox residence times were right skewed and log_10_ transformed for the analysis. We centered the binary predictor variables (open or half‐open vegetation structure, edge or noneedge location) and scaled the continuous predictor variables by dividing their means by two standard deviations (Gelman, 2008). The data points of the standardized predictor variables were within two standard deviations from the mean. We verified the assumptions by visual inspection and plotted the residuals against the predicted values. Finally, we corrected for the false discovery rate in each dataset by a simple sequential Bonferroni‐type procedure (Benjamini & Hochberg, 1995).

## 4 RESULTS

### 4.1 Effects of predator and habitat on prey residence time

**Figure 3:**
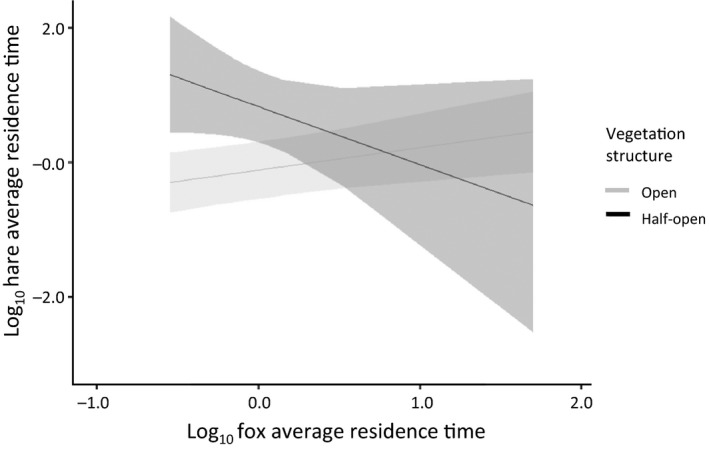
Hare average residence time ($\overset{¯}{X}\pm 95\%\text{CI}$) as a determinant of the interaction between the fox average residence time and vegetation structure (18 cameras, 186 visits, standardized). Rabbits were not detected by cameras

The presence of foxes affected the average residence time of hares in habitats that differed in shrub height, open or half‐open vegetation structure, and edge or noneedge location (Table 2, compare dataset A and C). Hares spent less time in habitats with low shrub height, open vegetation structure, or habitats away from edges. Additionally, hare average residence time was negatively related to fox average residence time in areas with a half‐open vegetation structure (Figure 3), whereas it was unrelated in areas with an open vegetation structure. The presence of foxes did not affect the average residence time of rabbits, although in the absence of foxes, rabbits spent more time in half‐open vegetation structures compared to open vegetation structures (Table 3, compare dataset E and F). Residence time of foxes was only correlated with edge habitat when hares were present. When hares were present, foxes spent more time in edge habitats compared to nonedge habitats (*t* = 4.9, *n* = 36, *p* < 0.0001).

**Table 2:** Conditional beta coefficients and adjusted standard errors of the standardized model parameters for models of the European hare average residence time related to species presence at trap camera locations

Trap camera dataset^a^		Hare (A)			Hare + rabbit (B)			Hare + fox (C)			Hare + rabbit + fox (D)		
No.	Model	Beta ± *SE* b	*t*	*p* c	Beta ± *SE*	*t*	*p*	Beta ± *SE*	*t*	*p*	Beta ± *SE*	*t*	*p*
1	MSH	<−0.1 ± 0.2	<0.01		0.4 ± 0.3	1.2		0.5 ± 0.2	2.8	*	0.1 ± 0.2	0.4	
2	RAB +				d						0.4 ± 0.2	2.6	
	MSH +				—						<−0.1 ± 0.2	0.2	
	RAB*MSH				—						−0.9 ± 0.2	3.5	**
3	FOX +							0.2 ± 0.2	1.0		0.2 ± 0.2	1.1	
	MSH +							0.5 ± 0.2	3.5	**	0.1 ± 0.2	0.5	
	FOX*MSH							−0.6 ± 0.6	1.0		0.4 ± 0.7	0.6	
4	OHO^e^	0.1 ± 0.2^f^	0.3		−0.9 ± 0.2	3.7	**	0.8 ± 0.2	3.9	**	−0.3 ± 0.2	1.5	
5	RAB +				−0.7 ± 0.2	3.0	*				0.4 ± 0.2	2.0	
	OHO +				−1.2 ± 0.2	6.1	***				−0.4 ± 0.2	1.9	
	RAB*OHO				1.4 ± 0.5	2.9	*				0.2 ± 0.5	0.4	
6	FOX +							0.1 ± 0.1	1.2		0.6 ± 0.2	2.9	
	OHO +							0.9 ± 0.1	6.4	***	0.1 ± 0.2	0.6	
	FOX*OHO							−1.2 ± 0.4	2.7	*	1.8 ± 0.6	3.2	**
7	POS^g^	0.1 ± 0.2	0.5		h			0.7 ± 0.3	2.5	*	0.2 ± 0.2^f^	1.0	
8	RAB				−0.1 ± 0.4	0.1					0.4 ± 0.2	1.8	
9	FOX							0.3 ± 0.2	1.4		0.2 ± 0.2	1.1	
10	RAB +										0.3 ± 0.2	1.2	
	FOX +										0.3 ± 0.2	1.6	
	RAB*FOX										−0.7 ± 0.6	1.1	

Note

FOX: predator average residence time; MSH: mean shrub height; OHO: open or half‐open vegetation structure; POS: nonedge or edge location; RAB: fellow prey average residence time.

a Trap camera dataset (cameras, number of visits): A (21, 71), B (8, 258), C (18, 186), D (20, 1,065).

b Beta's standardized by 2*SD (Gelman, 2008). The beta of an interaction is different in the slope between the two values when the covariate increases by 1 standard deviation.

c *p < 0.05, **p < 0.01, ***p < 0.001, significance level *α* is corrected for the false discovery rate in each dataset (Benjamini & Hochberg, 1995).

d Highly correlated fixed effects, *r* > 0.7.

e Open vegetation structure is the reference category.

f High rate of type II error because of unequal and small group sizes; ratio *n*_small_/*n*_large_ ≤ 0.5 (Supporting Information Table S3).

g Nonedge location is the reference category.

h Too few samples in nonedge locations to estimate the effect.

**Table 3:** Conditional beta coefficients and adjusted standard errors of standardized model parameters for models of the European rabbit average residence time related to species presence at trap camera locations

Trap camera dataset^a^		Rabbit (E)			Rabbit + hare (B)			Rabbit + fox (F)			Rabbit + hare + fox (D)		
No.	Model	Beta ± *SE* b	*t*	*p* c	Beta ± *SE*	*t*	*p*	Beta ± *SE*	*t*	*p*	Beta ± *SE*	*t*	*p*
1	MSH	0.1 ± 0.2	0.4		−0.6 ± 0.1	4.8	**	0.1 ± 0.1	0.5		−0.1 ± 0.2	0.4	
2	HAR +				0.2 ± 0.1	2.5					0.3 ± 0.1	2.5	
	MSH +				−0.8 ± 0.1	7.0	***				<−0.1 ± 0.1	0.2	
	HAR*MSH				0.5 ± 0.2	2.5					−0.8 ± 0.3	3.0	
3	FOX +							0.2 ± 0.2	1.1		−0.1 ± 0.2	0.3	
	MSH +							<0.1 ± 0.2	0.3		−0.1 ± 0.2	0.5	
	FOX*MSH							−0.1 ± 0.2	0.3		−0.4 ± 0.6	0.7	
4	OHO^d^	0.4 ± 0.2^e^	2.7	*	−0.5 ± 0.3	1.7		0.3 ± 0.1	2.5^e^		<0.1 ± 0.2	<0.1	
5	HAR +				<0.1 ± 2.6	<0.1					0.4 ± 0.2	2.7	
	OHO +				−0.5 ± 2.0	0.3					0.1 ± 0.2	0.6	
	HAR*OHO				0.9 ± 5.2	0.2					−0.5 ± 0.3	1.5	
6	FOX +							0.1 ± 0.2	0.5		0.1 ± 0.3	0.4	
	OHO +							0.3 ± 0.1	2.4		0.1 ± 0.3	0.4	
	FOX*OHO							0.1 ± 0.5	0.3^e^		0.6 ± 0.8	0.7	
7	POS^f^	0.3 ± 0.2^e^	2.1		g			0.4 ± 0.1	2.4^e^		−0.1 ± 0.2^e^	0.4	
8	HAR				0.1 ± 0.2	0.5					0.4 ± 0.2	2.7	
9	FOX							0.2 ± 0.1	1.2		−0.1 ± 0.2	0.3	
10	HAR +										0.4 ± 0.2	2.5	
	FOX +										−0.1 ± 0.2	0.8	
	HAR*FOX										−0.2 ± 0.5	0.4	

Note

FOX: predator average residence time; MSH: mean shrub height; OHO: open or half‐open vegetation structure; POS: nonedge or edge location; HAR: fellow prey average residence time.

a Trap camera dataset (cameras, number of visits): E (36, 222), B (8, 258), F (55, 995), D (20, 1,065).

b Beta's standardized by 2*SD (Gelman, 2008). The beta of an interaction is different in the slope between the two values when the covariate increases by 1 standard deviation.

c *p < 0.05, **p < 0.01, ***p < 0.001, significance level *α* is corrected for the false discovery rate in each dataset (Benjamini & Hochberg, 1995).

d Open vegetation structure is the reference category.

e High rate of type II error because of unequal group sizes; ratio *n*_small_/*n*_large_ ≤ 0.5 (Supporting Information Table S3).

f Nonedge location is the reference category.

g Too few samples in nonedge locations to estimate the effect.

### 4.2 Effects of fellow prey on predator–prey residence time

**Figure 4:**
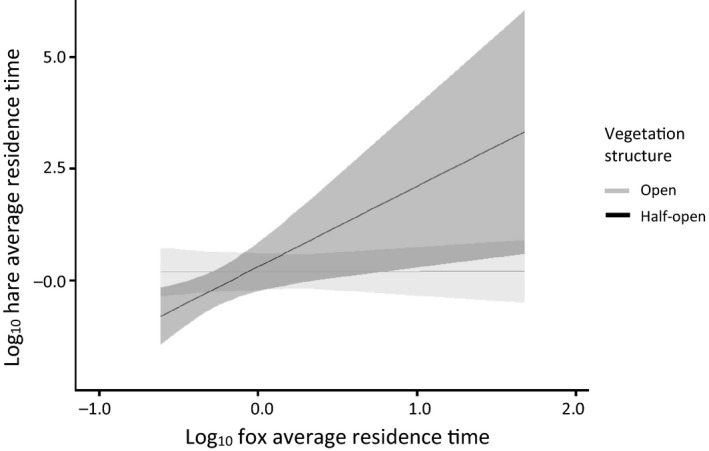
Hare average residence time ($\overset{¯}{X}\pm 95\%\text{CI}$) as a determinant of the interaction between the fox average residence time and vegetation structure (20 cameras, 1,065 visits, standardized). Rabbits were detected by cameras

The presence of rabbits (i.e., introducing shared predation) affected the relation between the residence times of foxes and hares. In the presence of rabbits, hare average residence time was positively related to fox average residence time in areas with a half‐open vegetation structure, whereas it was unrelated in areas with an open vegetation structure (Figure 4). This interaction was reversed in the absence of rabbits (see Figure 3; Table 2, compare dataset C and D). Additionally, when rabbits (and foxes) were present, hares did not avoid habitats with low shrub height, open vegetation structure, or habitats away from edges, comparable to locations where only hare was present (Table 2, compare dataset A, C, and D). The presence of hares did not affect the predator–prey space race between foxes and rabbits.

### 4.3 Effects of predator on fellow prey–prey residence time

**Figure 5:**
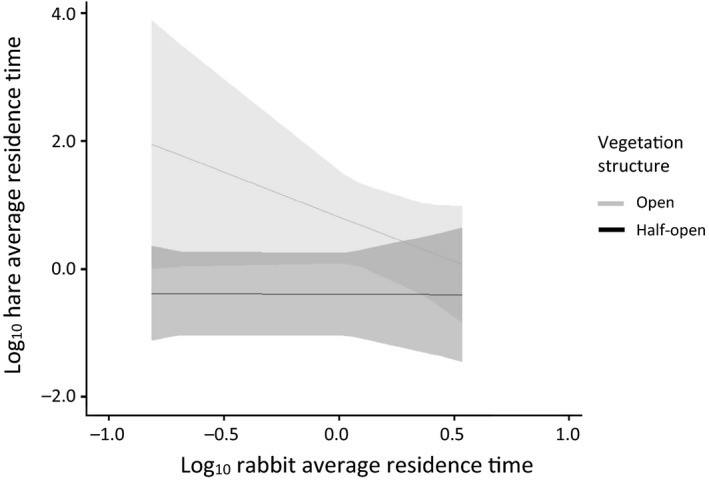
Hare average residence time ($\overset{¯}{X}\pm 95\%\text{CI}$) as a determinant of the interaction between the rabbit average residence time and vegetation structure (eight cameras, 258 visits, standardized). Foxes were not detected by cameras

The presence of foxes (i.e., introducing shared predation) affected the relation between the residence times of rabbits and hares. In the absence of foxes, hare average residence time was negatively related to rabbit average residence time in open vegetation structures, whereas it was unrelated in areas with a half‐open vegetation structure (Figure 5). However, hare average residence time and rabbit average residence time were unrelated in open or half‐open vegetation structures when foxes were present (Table 2, compare dataset B and D). In the absence of foxes (but presence of hares), rabbit average residence time was negatively related to shrub height, whereas rabbit average residence time and shrub height were unrelated when foxes (and hares) were present (Table 3, compare dataset B and D).

## 5 DISCUSSION

### 5.1 Prey and predator distribution among habitats differing in habitat riskiness

Overall, predator space use and habitat riskiness more strongly affected space use by hares than space use by rabbits. Noncentral‐place foragers like hares are more capable of shifting their use of space as a response to a change in predation risk, as they have larger home ranges, have access to a wider range of food resources, and possess multiple escape modes (Stott, 2007; Wirsing et al., 2010). Moreover, hares have a relatively small digestive system, which acts as a weight‐minimizing adaptation to enhance flight and maximizes the passage rate to cope with low‐quality forage (Kuijper et al., 2004; Stott, 2007). Therefore, hares can compensate for a poorer diet that comes at the cost of a shift in space (Laundré, Hernández, & Ripple, 2010). As a central‐place forager, rabbits are less capable of shifting their use of space as a result of predation risk. Even though predation risk is predicted to increase with distance from the central location of central‐place foragers (Lima & Dill, 1990), space use by a central‐place forager like rabbit was not affected by predation risk; instead space use was strongly affected by food quality (Bakker et al., 2005). However, rabbit space use could be affected by the spatial arrangement of their refuges (Wilson, Rayburn, & Edwards, 2012). Central‐place foragers should maximize net energy gain and have more difficulty to compensate for a poorer diet that comes at the cost of a shift in space (Demment & Van Soest, 1985; Schoener, 1979; Shipley, 2007; Stott, 2007). Hence, as a response to predation risk, central‐place foragers could shift their activities in time (Bakker et al., 2005).

As expected, hares spent more time in locations with tall shrubs and half‐open vegetation structures when foxes were present. Structure‐rich tall shrub and half‐open vegetation may reduce risk for prey that hide from predators by a reducing their encounter rate with predators (Lima & Dill, 1990; Riginos & Grace, 2008; Verdolin, 2006). However, hares also spent more time in locations near edges when foxes were present, even though foxes prefer protective cover, and ecotones, and are considered ambush predators (Holley, 1993; Kiener & Zaitsev, 2010). Prey escape mode is context dependent (Kuijper et al., 2015; Wirsing et al., 2010). Even though hares use closed vegetation as a hiding place (Neumann et al., 2011), and can conceal themselves from predators by their cryptic coloration (Focardi & Rizzotto, 1999), they could retain the option of flight by hiding in edge habitat. Possibly hares did not prefer to use open vegetation structures away from edges, because habitat riskiness of open vegetation structures in a mosaic of vegetation patches may depend on landscape characteristics, such as the patch size of the vegetation that can affect the probability of attack and the probability of escape (Heithaus, Wirsing, Burkholder, Thomson, & Dill, 2009).

### 5.2 The effects of shared predation on space use by prey

Few field studies on terrestrial systems have quantitatively investigated the effects of shared predation on fine‐scale space use by prey species (Camacho et al., 2017; Johnson et al., 2013; Oliver et al., 2009). As expected, the presence of rabbits (i.e., introducing shared predation) affected the predator–prey space race for hares more strongly than vice versa. However, we did not find shared predation to negatively affect space use by hare as expected. In contrast, we found that shared predation reduced predation risk for hares and did not affect rabbit. Fellow prey that are preferred prey, such as rabbit, can attract predators (Díaz‐Ruiz et al., 2011; Doherty et al., 2015; Norbury, 2001; Smith & Quin, 1996) that can reduce the probability of being targeted or increase the probability of escape for other prey when collectively detected (Bednekoff & Lima, 1998). Predation risk for the weaker competitor is expected to be lower, if the stronger competitor is more vulnerable to predation (Grand & Dill, 1999). Both prey are then expected to aggregate in the risky but productive open habitat. This corroborates our results, particularly, if we assume that the smaller rabbits have an exploitative competitive advantage over the larger hares (Flux, 2008; Persson, 1985; Shipley, 2007), and that rabbits are more vulnerable to predation because they are preferred prey of predators like feral cat and red fox. Preferred prey species experience regulatory predation, in contrast to prey species that are less preferred, which are more prone to depensatory predation (DeCesare et al., 2010; Sinclair et al., 1998).

Hares and rabbits seemed to be negatively associated in open vegetation structure and in tall shrubs in the absence of foxes. This behavior could erroneously be interpreted as apparent competition, but in our case, this is most likely the result of exploitative competition (Halliday & Morris, 2013). However, exploitative and apparent competition can act simultaneously between sympatric species (Chase et al., 2002; Noonburg & Byers, 2005). The presence of foxes (i.e., introducing shared predation) affected the space use between hares and rabbits. The presence of a shared predator can release competition between prey species and promote their coexistence in productive open vegetation structures with high risk, and in landscapes that contain spatial variation in habitat riskiness (Bonsall, Bull, Pickup, & Hassell, 2005; Bonsall & Hassell, 2000; DeCesare et al., 2010; Grand & Dill, 1999; Gurevitch, Morrison, & Hedges, 2000). Nevertheless, it seemed that even though foxes were present, the negative association between hares and rabbits in tall shrubs with low risk persisted (see Table 2 dataset D), possibly because rabbit as a stronger competitor prefers burrows in edge habitat with protective shrub cover (Bakker et al., 2005).

Several other factors could explain the response of prey to shared predation. First, the presence and density of other prey species, such as mice, and ground breeding birds will affect the relationship between our prey and their shared predator, because additional prey can affect the density of predators (Carbone & Gittleman, 2002; Duffy et al., 2007). Nevertheless, in our coastal dune landscape, a small part of the fox diet (<25% of feces content) did not constitute of rabbit and hares (Mulder, 2005). Second, the timing of prey responses to predation risk is important (Lima & Dill, 1990). Prey species can vary in their space use over time and can shift their space use in time as a response to increased predation risk (Eccard, Pusenius, Sundell, Halle, & Ylönen, 2008; Tambling et al., 2015), affecting the temporal overlap between prey and predator (Linkie & Ridout, 2011). Third, the prey space use is affected by the trade‐off between food and the risk of predation that is related to prey body size and food availability (Hopcraft, Anderson, Pérez‐Vila, Mayemba, & Olff, 2012; Hopcraft, Olff, & Sinclair, 2010; Owen‐Smith, Fryxell, & Merrill, 2010). Compared to hare, rabbit is much smaller in body size. Space use by rabbits is thus more strongly affected by food than predation (Bakker et al., 2005; Hopcraft et al., 2012, 2010), because the metabolic rate of smaller prey is relatively higher, mass‐specific nutritional requirements increase with declining body mass, and smaller herbivorous mammals are more limited in their digestive efficiency (Demment & Van Soest, 1985; Owen‐Smith, 1988; Schmidt‐Nielsen, 1990). Possibly, rabbits are thus unable to avoid predation risk via spatial shifts, because of their foraging mode as a result of their food requirements and the central place of their burrow.

## 6 CONCLUSIONS

Hares and rabbits spent time differently among habitats that differ in riskiness. Space use by predators and habitat riskiness affected space use by hares as a noncentral‐place forager more strongly than space use by rabbits as a central‐place forager. Residence time of hare was shorter in habitats in which the predator was efficient in searching or capturing prey species. However, hares spent more time in edge habitat when foxes were present, even though foxes are considered ambush predators. Habitat riskiness and species presence interacted with the space use by predators and the space use by fellow prey on the space use by prey. Shared predation affected the predator–prey space race for hares positively, and more strongly than the predator–prey space race for rabbits, which were not affected. Shared predation reversed the predator–prey space race between foxes and hares, whereas shared predation possibly also released a negative association and promoted a positive association between hares and rabbits. Prey species’ properties, such as escape mode and characteristics that affect foraging mode (i.e., body size, competitive ability, and dependence or independence of a prey to a central place), affect the space use by prey among habitats that differ in riskiness by shared predation.

## CONFLICT OF INTEREST

The authors declare they have no conflict of interest.

## AUTHOR CONTRIBUTIONS

Conception and design: MW, SE, JP, HK, PJ, and SvW, acquisition of data: MW, SE, and JP, analysis and interpretation of data: MW, HK, PJ, and FvL, manuscript: MW, DK, HP, PJ, FvL, and SvW wrote the manuscript. All authors read, reviewed, and approved the final manuscript.

## Data Availability

The data are deposited in the Dryad Digital Repository.
