# Supplementary material for: Implications of shared predation for space use in two sympatric leporids
Source: Ecol Evol. 2019 Feb 20;9(6):3457–69. doi: 10.1002/ece3.4980 (PMC6434570; doi:10.1002/ece3.4980)
Supplement: Supplementary file 1 [file ECE3-9-3457-s001.docx]

**Table S1:** An overview of the vegetation types of camera locations in the study area.

| No. | Vegetation types ^1^ | # cameras |
| --- | --- | --- |
| 1 | Calcareous dune grassland | 66 |
| 2 | Dune grasslands | 44 |
| 3 | Burnet rose, creeping willow-, blackberry thicket | 24 |
| 4 | Flower rich grasslands | 21 |
| 5 | Thickets | 6 |
| 6 | Deciduous forest | 4 |
| 7 | Near-shore communities | 4 |
| 8 | Calcareous dune valley | 2 |
| 9 | Nutrient rich grasslands | 2 |
| 10 | Non-calcareous dune grassland | 1 |
| 11 | Remaining forest | 1 |
| 12 | Reed swamp | 1 |
| 13 | Reed swamp communities | 1 |
|  | total | 177 |

^1^ Based on an overlay between the camera locations and a high resolution (1:5000) GIS vegetation map by Everts et al. (2008; 2009).

**Table S2:** Effective detection distance, angle and average geometric speed $\left( \bar{X}\pm s.e. \right)$ of European hare (*Lepus europeus*), European rabbit (*Oryctolagus cuniculus*) and fox (*Vulpes vulpes*) in 8 habitat types of the dune landscape.

| **Vegetation structure** | **Edge**  **type** | **Effective detection distance** (m) | | | **Effective detection angle** (degrees) | | | **Average geometric speed** (cm/s) | | |
| --- | --- | --- | --- | --- | --- | --- | --- | --- | --- | --- |
|  |  | **Hare** | **Rabbit** | **Fox** | **Hare** | **Rabbit** | **Fox** | **Hare** | **Rabbit** | **Fox** |
|  |  | N = 163 | N = 41 | N = 174 | N =163 | N = 41 | N = 174 | N = 149 | N = 40 | N = 165 |
| Open | non-edge | 6.0 ± 0.3 | 7.1 ± 0.5 | 5.4 ± 0.2 | 14 ± 1 | 14 ± 2 | 14 ± 1 | 15 ± 2 | 6 ± 3 | 43 ± 2 |
|  | edge half-open |  |  |  |  |  |  | 25 ± 3 | 10 ± 3 | 74 ± 2 |
|  | edge forest |  |  |  |  |  |  | 15 ± 2 | 7 † | 60 ± 2 |
|  | edge thicket |  |  |  |  |  |  | 24 ± 3 | 10 † | 54 ± 2 |
|  |  | N = 40 | N = 40 | N = 168 | N = 40 | N = 40 | N = 168 | N = 35 | N = 40 | N = 157 |
| Half-open | non-edge | 4.1 ± 0.4 | 3.9 ± 0.4 | 4.7 ± 0.2 | 19 ± 2 | 18 ± 1 | 21 ± 1 | 7 ± 7 | 3 ± 3 | 32 ± 2 |
|  | edge open |  |  |  |  |  |  | 5 ± 3 | 2 ± 3 | 24 ± 3 |
|  | edge forest |  |  |  |  |  |  | 14 ± 3 | 6 † | 31 ± 2 |
|  | edge thicket |  |  |  |  |  |  | 35 ± 4 | 15 † | 39 ± 2 |

† estimated based on the ratio between the average geometric speed by hares in the vegetation structures

**Table S3:** An overview of the characteristics of the response and predictor variables used in the datasets.

| Trap camera dataset ^†^ | | A | B | C | D | E | F |
| --- | --- | --- | --- | --- | --- | --- | --- |
| Sample size (# cameras) | | 21 | 8 | 18 | 20 | 36 | 55 |
| Uncorrected average time spent in front of the camera, averaged over all cameras in the dataset (sec) | Hare | 18.8 ± 12.2 ^‡^  5.0 – 46.3 | 9.8 ± 7.5  5.0 – 24.0 | 21.1 ± 17.7  6.0 – 69.0 | 11.7 ± 7.6  5.0 – 33.3 |  |  |
|  | Rabbit |  | 19.3 ± 12.9  5.0 – 39.3 |  | 28.5 ± 28.3  5.0 – 126.2 | 31.2 ± 37.9  1.0 – 186.8 | 22.6 ± 15.1  5.0 – 61.8 |
| Average patch residence time, averaged over all cameras in the dataset (sec/hrs*m^2^) | Hare | 4.9 ± 5.0  0.8 – 20.5 | 1.8 ± 1.8  0.6 – 5.4 | 5.9 ± 7.6  1.0 – 33.3 | 2.7 ± 1.6  0.7 – 5.9 |  |  |
|  | Rabbit |  | 2.7 ± 1.5  0.7 – 4.9 |  | 4.9 ± 3.6  0.6 – 15.6 | 15.6 ± 35.7  0.1 – 184.3 | 8.8 ± 12.3  0.7 – 60.9 |
| Uncorrected total time spent in front of the camera, averaged over all cameras in the dataset (sec) | Hare | 77.4 ± 122.8  5.0 – 576.0 | 51.9 ± 105.4  5.0 – 311.0 | 133.6 ± 171.0  6.0 – 593.0 | 59.2 ± 63.5  5.0 – 200.0 |  |  |
|  | Rabbit |  | 669.9 ± 1129.0  5.0 – 3201.0 |  | 1769.8 ± 2893.7  10.0 – 9938.2 | 193.4 ± 281.1  1.0 – 1121.0 | 440.3 ± 731.9  5.0 – 4756.0 |
|  | Fox |  |  | 43.1 ± 42.9  5.0 – 144 | 32.0 ± 77.8  5.0 – 357.4 |  | 48.0 ± 166.6  3.0 – 1240.2 |
| Total patch residence time, averaged over all cameras in the dataset (sec/hrs*m^2^) | Hare | 16.4 ± 21.7  0.8 – 95.1 | 12.8 ± 29.7  0.6 – 86.3 | 34.0 ± 41.6  1.0 – 133.1 | 13.0 ± 14.5  0.7 – 49.2 |  |  |
|  | Rabbit |  | 95.4 ± 151.1  0.7 – 394.7 |  | 310.6 ± 516.8  1.3 – 1906.0 | 96.8 ± 229.3  0.1 – 1105.7 | 220.3 ± 656.3  0.8 – 4691.1 |
|  | Fox |  |  | 11.6 ± 11.5  1.0 – 39.6 | 10.1 ± 27.2  1.0 – 124.2 |  | 12.1 ± 39.3  0.5 – 291.3 |
| Mean shrub height (cm) |  | 21.2 ± 25.8  0.0 – 75.0 | 25.3 ± 21.9  0.0 – 52.3 | 21.9 ± 39.2  0.0 – 128.0 | 24.5 ± 24.5  0.0 – 85.0 | 50.1 ± 36.7  0.0 – 170 | 47.6 ± 32.2  0.0 – 135.0 |
| Vegetation structure  (# cameras) | Open | 14 ^§^ | 4 | 15 | 13 | 12 | 16 |
|  | Half-open | 7 | 4 | 3 | 7 | 24 | 39 |
| Risky places  (# cameras) | Non-edge | 12 | 1 | 4 | 5 | 11 | 17 |
|  | Edge | 9 | 7 | 14 | 15 | 25 | 38 |
| Minimum average distance between cameras (m) | Session  1  2  3  4  5 | 460 ± 502, 52, 6 ^¶^  1265 ± 2017, 212, 5  1802 ± 2465, 425, 4  2205 ± 2360, 843, 3  333 ± 387, 109, 3 | - ± -, -, 1    1315 ± 0, 1315, 2  - ± -, -, 1  1185 ± 974, 385, 4 | 1460 ± 2715, 82, 4    471 ± 220, 344, 3  1202 ± 1983, 184, 4  759 ± 682, 178, 7 | 5580 ± 0, 5580, 2  - ± -, -, 1  2604 ± 2651, 1073, 3  505 ± 444, 50, 6  596 ± 1260, 98, 8 | 456 ± 280, 173, 8  295 ± 184, 92, 11  395 ± 242, 135, 6  672 ± 873, 205, 8  - ± -, -, 1 | 378 ± 383, 117, 11  293 ± 242, 26, 11  232 ± 165, 70, 14  373 ± 360, 85, 8  162 ± 228, 4, 11 |

^†^ Trap camera dataset = species involved: A = hare, B = hare & rabbit, C = hare & fox, D = hare, rabbit & fox, E = rabbit, F = rabbit & fox;

^‡^ continuous variables: Χ ± SD, Min – Max; ^§^ binary variables: number of cameras, ^¶^ minimum average distance between cameras: Χ ± SD, Min, number of cameras.
